# Functional dependence of resonant harmonics on nanomechanical parameters in dynamic mode atomic force microscopy

**DOI:** 10.3762/bjnano.8.90

**Published:** 2017-04-19

**Authors:** Federico Gramazio, Matteo Lorenzoni, Francesc Pérez-Murano, Enrique Rull Trinidad, Urs Staufer, Jordi Fraxedas

**Affiliations:** 1 Catalan Institute of Nanoscience and Nanotechnology (ICN2), CSIC and The Barcelona Institute of Science and Technology, Campus UAB, Bellaterra, 08193 Barcelona, Spain; 2 Instituto de Microelectrónica de Barcelona (IMB-CNM, CSIC), Campus UAB, 08193 Bellaterra, Barcelona, Spain; 3 Technical University of Delft, Mekelweg 2, 2628CD Delft, The Netherlands

**Keywords:** atomic force microscopy, metrology, multifrequency, nanomechanics

## Abstract

We present a combined theoretical and experimental study of the dependence of resonant higher harmonics of rectangular cantilevers of an atomic force microscope (AFM) as a function of relevant parameters such as the cantilever force constant, tip radius and free oscillation amplitude as well as the stiffness of the sample’s surface. The simulations reveal a universal functional dependence of the amplitude of the 6th harmonic (in resonance with the 2nd flexural mode) on these parameters, which can be expressed in terms of a gun-shaped function. This analytical expression can be regarded as a practical tool for extracting qualitative information from AFM measurements and it can be extended to any resonant harmonics. The experiments confirm the predicted dependence in the explored 3–45 N/m force constant range and 2–345 GPa sample’s stiffness range. For force constants around 25 N/m, the amplitude of the 6th harmonic exhibits the largest sensitivity for ultrasharp tips (tip radius below 10 nm) and polymers (Young’s modulus below 20 GPa).

## Introduction

When an AFM cantilever oscillating freely and harmonically at a given frequency *f* and amplitude *A*_1_ approaches a solid surface, the oscillation becomes anharmonic due to the non-linear interaction, represented by the force field *F*_ts_, between the cantilever tip and the surface [1]. Thus, the time dependent trajectory *a*(*t*) of the cantilever tip, which can be expressed in the harmonic limit by *a*(*t*) = *A*_1_cos(2π*ft*), is transformed into a Fourier series with harmonic oscillations of amplitudes *A_n_* and frequencies *f_n_* = *nf* [2–3]:

[1]
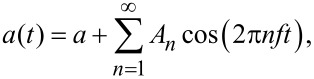


where *a* is a constant amplitude value. The *A_n_* coefficients can be analytically calculated, in the limit of weak tip–sample interaction (*A*_1_ >> *A_n_* for *n* > 1), by integrating the force field *F*_ts_ that is modulated by weighted Chebyshev polynomials of the first kind, *T_n_*(*u*) [4–5]. The simplified expression is:

[2]
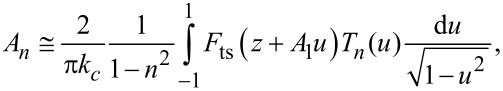


where *k*_c_ stands for the cantilever stiffness, *z* is the distance between the cantilever base and the sample surface and *T_n_*(*u*) = cos(*n*cos^−1^(*u*)). Note that *A_n_* decreases for increasing order number (*n*) and *k*_c_ values.

The tip–surface interaction *F*_ts_ can be expressed as a function of experimental parameters, such as the tip radius (*R*) and the Young’s modulus of the sample (*E*). A list of well-accepted models can be found in the literature, including the most widely used Hertz, Derjaguin–Muller–Toporov (DMT) and Johnson–Kendall–Roberts (JKR) models, describing the analytical dependence on such parameters [6–10]. The DMT model, which will be used in this work, has the following expression in the repulsive regime:

[3]
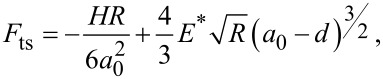


where *H* is the Hamaker constant, *a*_0_ the intermolecular distance, *d* the tip–sample gap (related to *z*) and E* the reduced Young modulus, which includes the contribution from the tip. Thus, by combining Equation 2 and Equation 3 it is possible to determine the dependence of *A_n_* as a function of all relevant parameters. However, *A_n_* can be hardly solved analytically, so that numerical simulations are required. On the other hand, the dynamics of the oscillating cantilever cannot be oversimplified and flexural eigenmodes must be considered, in particular when they are located close to one of the harmonics [11].

Here, we report a combined theoretical and experimental study on the functional dependence of the amplitudes of higher harmonics on relevant parameters such as the tip radius, free oscillation amplitude, cantilever stiffness and sample Young’s modulus. Because of the low amplitudes of the involved harmonics (well below 1 nm), we concentrate on the repulsive regime of the tip–sample interaction and on those harmonics close to flexural eigenmodes of rectangular cantilevers, hence the term resonance, so that their intensities can be reliably determined [11–12]. Previous results have shown that the intensity of the 6th harmonic is noticeably larger that the intensities of the neighbouring 5th and 7th harmonics, respectively, using cantilevers with 350 kHz resonant frequency and polystyrene samples [13]. Such study has provided a practical qualitative method to continuously monitor changes in the shape and radius of a cantilever tip in amplitude modulation AFM mode. Here, we will also focus on the 6th harmonic since it provides the larger amplitude, but the methodology can now be extended to other harmonics.

The work presented here provides a tool to help to prepare experiments. Such tool is a mathematical expression describing the evolution of the 6th harmonic on experimental parameters such as tip radius, free amplitude, cantilever and sample’s stiffness. For given experiments directed to, e.g., the determination of the evolution of the tip radius evolution and of sample stiffness, the mathematical tool should help in the selection of the favourable range of parameter values (including amplitude setpoint).

## Results and Discussion

### Simulations

**Figure 1:**
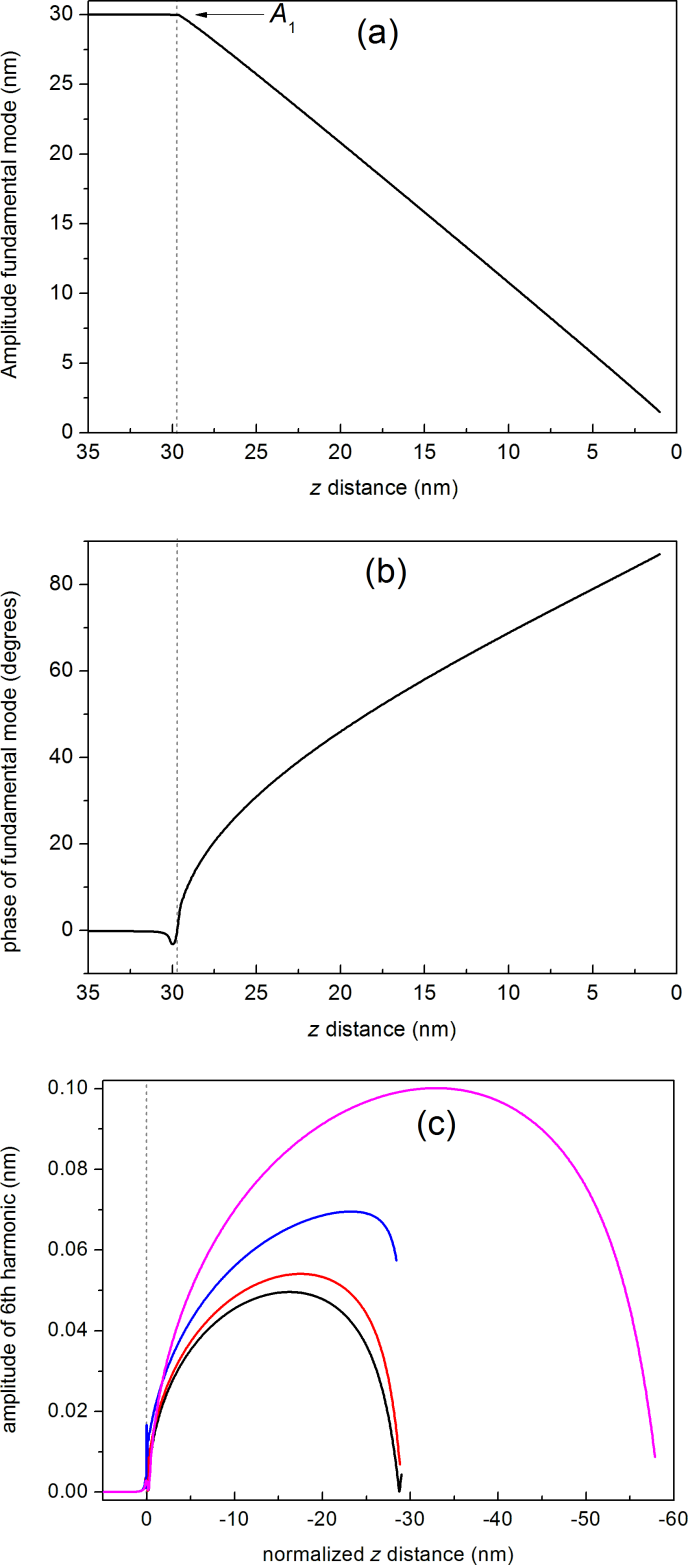
(color online) Simulated approach curves: (a) amplitude of the fundamental mode, (b) phase of the fundamental mode and (c) amplitude of the 6th mode as a function of the normalized *z* distance. The calculations have been performed for a silicon rectangular cantilever with *k*_c_ = 25 N/m, *f*_0_ = 300 kHz and *Q* = 400. The following parameters have been used: (a) and (b) *A*_1_ = 30 nm, *R* = 10 nm, *E* = 3 GPa, (c) continuous black curve *A*_1_ = 30 nm, *R* = 10 nm, *E* = 3 GPa; continuous red curve *A*_1_ = 30 nm, *R* = 30 nm, *E* = 3 GPa; continuous blue curve *A*_1_ = 30 nm, *R* = 10 nm, *E* = 130 GPa; continuous magenta curve *A*_1_ = 60 nm, *R* = 10 nm, *E* = 3 GPa.

Let us analyse first the dependence of the amplitude of the 6th harmonic, *A*_6_, on the independent parameters *R*, *E* and *A*_1_, respectively, for a fixed *k*_c_ value. We will analyse the dependence of *A*_6_ on *k*_c_ at a later stage. Note that *A*_1_ can be externally and continuously varied by selecting the excitation amplitude and frequency of the cantilever base and that *R* and *E* depend both on the materials used. Figure 1a and Figure 1b show the calculated approach curves (as a function of *z*) corresponding to the amplitude and phase lag φ of the fundamental mode, respectively, for *A*_1_ = 30 nm, *R* =10 nm and *E* = 3 GPa using a 25 N/m silicon cantilever with a resonant frequency *f*_0_ = 300 kHz and a *Q* factor of 400. The threshold of the repulsive region (φ > 0 degrees) is represented by a vertical discontinuous grey line. Figure 1c displays the *A*_6_ approach curves corresponding to the parameters used in Figure 1a and Figure 1b (continuous black line), as well as those obtained by increasing *R* (continuous red line), *E* (continuous blue line) and *A*_1_ (continuous magenta line), respectively. The black line shows the shape of the *A*_6_(*z*) curve, with *A*_6_ > 0 above the repulsive regime threshold, exhibiting a maximum value at *z* ≈ *A*_1_/2 and decreasing back to zero for sufficiently small *z* values, which correspond to small oscillation amplitudes, as depicted from Figure 1a.

From Figure 1c we readily observe that *A*_6_ increases for increasing *R*, *E* and *A*_1_ values at a given *z* value, respectively. When *R* increases (continuous black and red curves) we observe, in addition, that the maximum of the curve shifts towards lower *z* values, a behaviour that is also observed for increasing *E* values (compare continuous black and blue lines). In fact the weight of the area under the curves is shifted towards lower *z* values in both cases.

The comparison between the continuous black and red curves indicates that the variation of the radius has an impact in the value of *A*_6_ only for certain values of the amplitude setpoint (*A*_sp_): for example at *A*_sp_ = 25 nm (*z* = 24.2 nm) the difference between the black and red curves is clearly smaller than for *A*_sp_ = 15 nm (*z* = 14.2 nm). The same argument applies for the case of increasing *E* values. It means that the previous modelling of the system is necessary in order to select the appropriate experimental conditions.

**Figure 2:**
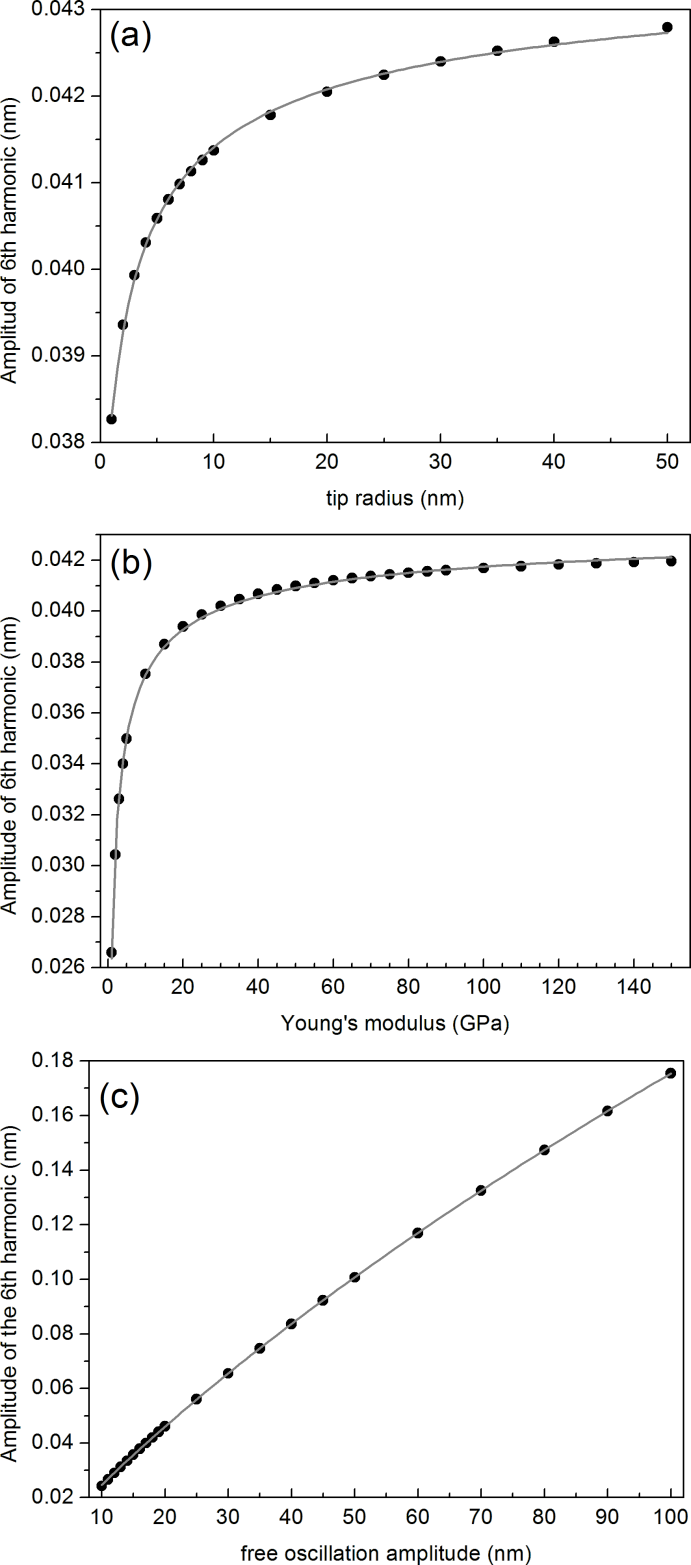
Simulated dependence of the amplitude of the 6th harmonic as a function of: (a) tip radius, (b) sample’s Young’s modulus and (c) free oscillation amplitude, respectively. The calculated points (filled black circles) have been performed for *k*_c_ = 26 N/m, *f*_0_ = 300 kHz and *Q* = 400 with *A*_sp_ = 0.5*A*_1_ and *A*_1_ = 20 nm for (a) and (b) and have been fitted to the gun-shaped function *G* described in Equation 4. The values of the *g*_1_, *g*_2_ and *g*_3_ parameters obtained from the fits are: (a) *g*_1_ = −1.63, *g*_2_ = 22.727 nm^−1^, *g*_3_ = 5.007 nm^−1/2^, (b) *g*_1_ = 4.373 GPa·nm^−1^, *g*_2_ = 22.84 nm^−1^, *g*_3_ = 10.737 GPa^1/2^·nm^−1^ and (c) *g*_1_ = 338.23, *g*_2_ = 0.285 nm^−1^, *g*_3_ = 20.325 nm^−1/2^.

Let us now explore in detail the dependence of *A*_6_ as a function of *R*, *E* and *A*_1_. The corresponding curves are shown in Figure 2, where the filled black points correspond to calculated values and the continuous lines to least-square fits. The simulations have been performed for silicon with the following parameters: *k*_c_ = 26 N/m, *f*_0_ = 300 kHz and *Q* = 400, with *A*_sp_ = 0.5*A*_1_. All curves confirm the increase of *A*_6_ for increasing values of *R*, *E* and *A*_1_ but with different evolutions. In the case of the phase of the 6th harmonic the behaviour is opposite, i.e., it decreases for increasing *R* values (see Supporting Information File 1, Figure S1).

In all cases the points closely follow a gun-shaped function with the expression:

[4]
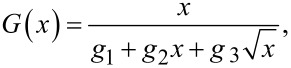


where *g*_1_, *g*_2_ and *g*_3_ are real numbers. This function covers the limiting cases of *G*(*x*) = *x*/*g*_1_ for *g*_1_ ≠ 0 and *g*_2_ = *g*_3_ = 0, *G*(*x*) = 1/*g*_2_ for *g*_2_ ≠ 0 and *g*_1_ = *g*_3_ = 0 and *G*(*x*) = √x/*g*_3_ for *g*_3_ ≠ 0 and *g*_1_ = *g*_2_ = 0. Figure 2c exemplifies the case where the dependence is nearly linear, an approximation certainly valid for smaller intervals around a central amplitude value. In the case of Figure 2a and Figure 2b we clearly observe two well defined regions corresponding to lower and higher *R* and *E* values, respectively. At lower values *A*_6_ exhibits a strong variation as a function of *R* and *E* (large slope) while at higher values *A*_6_ tends towards an asymptotic limit (small slope). Thus, for the *k*_c_ values used in this simulation (26 N/m) *A*_6_ is most sensitive for values of tip radius below ca.10 nm (ultrasharp tips) and sample’s Young’s modulus below ca. 20 GPa (typically polymers).

The dependence of *A*_6_ on the cantilever properties is more complex because the cantilever shape, the elastic constant *k*_c_, the resonance frequency *f*_0_ and the quality factor *Q* are interrelated parameters that all contribute to the dynamics of the oscillation. For simplicity, we characterize the cantilever by *k*_c_. Sader’s formula, also termed Sader’s method, gives a well-accepted expression of the dependence of *k*_c_ on the other parameters [14]:

[5]
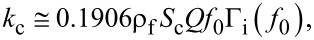


where ρ_f_ is the density of the fluid (in our case air), *S*_c_ is the plan view area of the cantilever (width × length) and Γ_i_ the imaginary component of the hydrodynamic function [15]. This expression is valid for *Q* >> 1.

**Figure 3:**
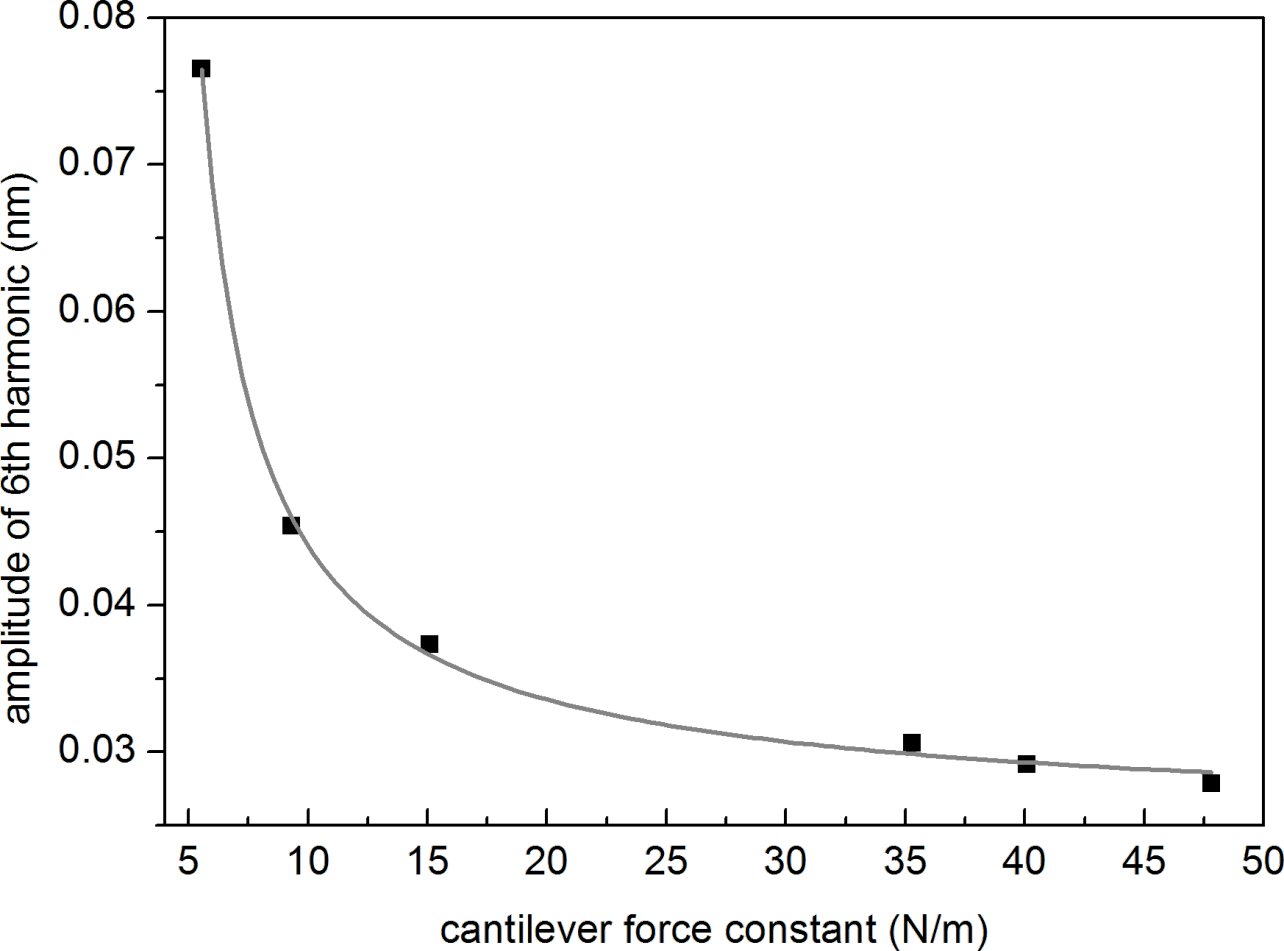
Simulated dependence of the amplitude of the 6th harmonic as a function of selected values of cantilever force constants. The chosen commercial cantilevers are: RTESP-150 (BRUKER) (*f* = 150 kHz, *L* = 125 μm, *W* = 35 μm), *Q* = 250, *k*_c_ = 5.5 N/m; 200AC-NA (μMASCH) (*f* = 150 kHz, *L* = 200 μm, *W* = 40 μm), *Q* = 330, *k*_c_ = 9.6 N/m; PPP-SEIH (NANOSENSORS) (*f* = 130 kHz, *L* = 225 μm, *W* = 33 μm), *Q* = 490, *k*_c_ = 15.1 N/m; RFESPA 190 (BRUKER) (*f* = 190 kHz, *L* = 225 μm, *W* = 40 μm), *Q* = 550, *k*_c_ = 35.3 N/m; RTESP-300 (BRUKER) (*f* = 300 kHz, *L* = 125 μm, *W* = 40 μm), *Q* = 570, *k*_c_ = 40.1 N/m; LTESP (BRUKER) (*f* = 190 kHz, *L* = 225 μm, *W* = 38 μm), *Q* = 590, *k*_c_ = 47.8 N/m.

In order to obtain an approximate manageable expression of the functional dependence of *A*_6_ on *k*_c_ we have used the nominal geometrical and resonance frequency values of different commercial rectangular cantilevers, as provided by the manufacturers and *Q* factor values in the range 200 ≤ *Q* ≤ 600. The resulting *k*_c_ values obtained from Equation 4 have been used as input to the VEDA code [16]. The result from this calculation is shown in Figure 3. The selected cantilevers with their values are shown in the caption to the figure.

The calculated points in the figure also follow quite closely Equation 4, with *g*_1_ = −63.092 nN·nm^−2^, *g*_2_ = 42.371 nm^−1^ and *g*_3_ = −42.223 nN^1/2^·nm^−3/2^.

Thus, the simulations provide a functional dependence of *A*_6_ on the relevant *R*, *E*, *A*_1_ and *k*_c_ parameters, which can be stated as:

[6]
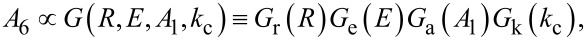


where *G*_r_, *G*_e_, *G*_a_ and *G*_k_ represent the decoupled gun-shaped functions with their corresponding coefficients *g*. The apparently different behaviours can be described by a single universal curve, where the magnitudes and signs of the *g* coefficients determine the final shapes. This expression can substantially simplify the analysis of partial contributions. In addition, it paves the way to define a methodology for finding the optimal experimental conditions to monitor or characterize a specific magnitude from the acquisition of the amplitude of a higher harmonic.

### Experiments

In the following section we compare the proposed functional dependence with experimentally derived results to validate the modelling and simulations described in previous sections.

#### Dependence of the amplitude of the 6th harmonic on tip radius

**Figure 4:**
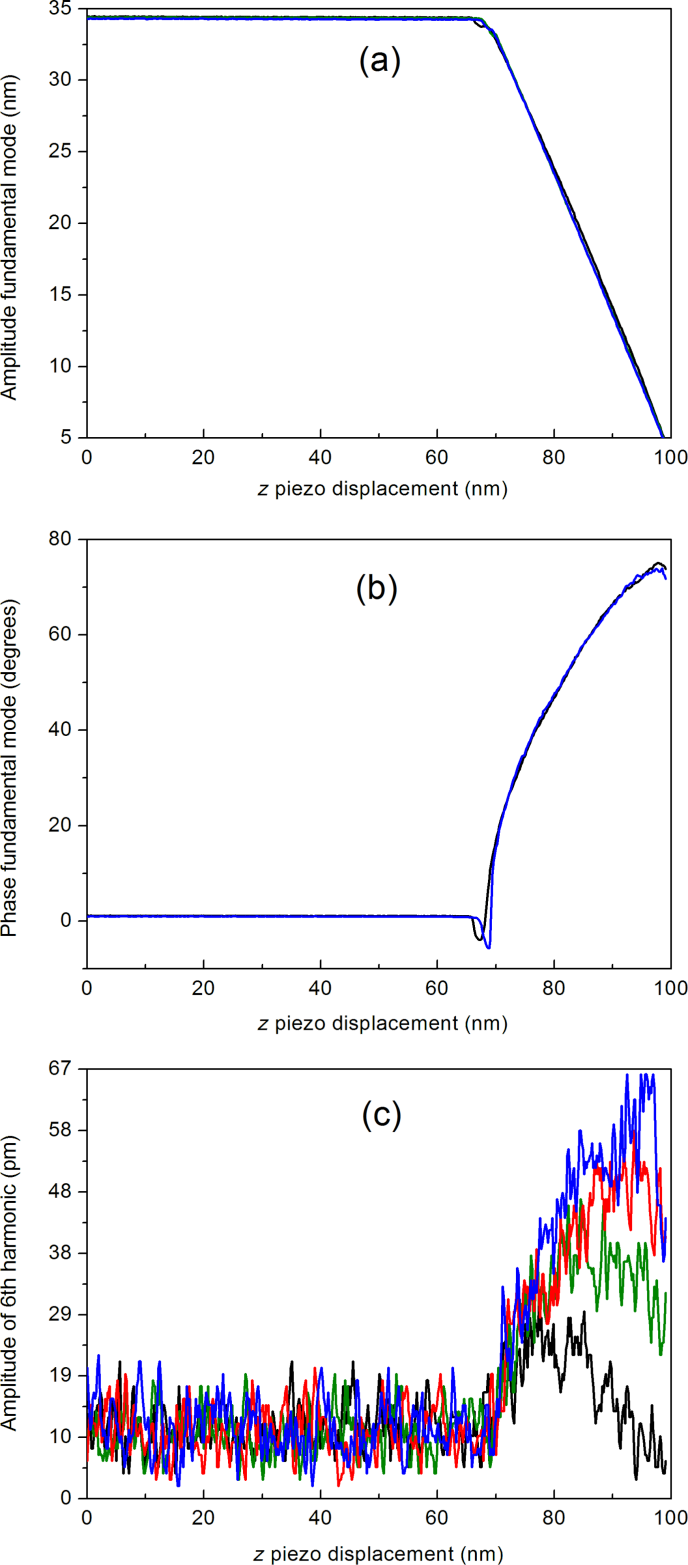
(color online) Approach curves taken alternatively with the acquisition of topographic, phase and amplitude images. (a) Amplitude of the fundamental mode, (b) phase of the fundamental mode and (c) amplitude of the 6th harmonic. Experiments have been performed with a nominally *k*_c_ ≈ 44 N/m rectangular AFM cantilever with *f*_0_ = 293 kHz on silicon surfaces and *A*_1_ = 34 nm.

Let us first explore the shape of the experimental approach curves and compare to the calculations shown in Figure 1. Figure 4 shows simultaneously acquired experimental approach curves, i.e., amplitude (a) and phase (b) of the fundamental mode and amplitude of the 6th harmonic (c), respectively, as a function of the piezo displacement in the direction perpendicular to the surface (*z*) for a nominally *k*_c_ ≈ 44 N/m cantilever with resonance frequency 293 kHz on silicon surfaces and *A*_1_ = 34 nm. The black curve in Figure 4c was taken at the beginning of the experiment (fresh tip). The rest of the curves are acquired after the acquisition of several intermediate images (i.e., several images are taken in between each approach curve). The order in which the curves are taken was black, green, red and blue, respectively.

The first conclusion that we can extract is that the simulated curves (Figure 1a and Figure 1b) reproduce well the shape of the experimental ones (Figure 4a and Figure 4b). In addition, Figure 4a and Figure 4b provide evidence that both the amplitude and phase of the fundamental mode remain unchanged during the experiments, except for a small variation in the phase at the transition between attractive to repulsive regimes (φ ≈ 0). Conversely, the value of *A*_6_ as a function of the piezoscanner displacement exhibits an increase over the noise level above the threshold corresponding to the onset of the repulsive regime. We clearly observe an increase of the maximum value of *A*_6_ as well as an increase of the position of the maximum, a trend that is reproduced by the simulations, as depicted from Figure 1c. Assuming that both sample’s Young’s modulus and *k*_c_ remain constant, then the increase of *A*_6_ can be attributed primarily to an increase in *R*.

The small variation of the phase (see Figure 4b) occurs at the transition between attractive to repulsive regimes, where the critical amplitude *A*_c_ is defined [17]. It turns out that *A*_c_ depends on *R*, closely following a power law function (*A*_c_ 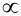 *R^m^*), where the parameter *m* (*m* < 1) depends on the particular cantilever used [17–20]. This provides a further evidence that the observed increase of *A*_6_ can be attributed to an increase in *R*. On the other hand, *A*_c_ is evaluated, in those works, at the sharp attractive–repulsive transition, which implies a rather involved experimental determination, while using approach curves one can select the setpoint and thus the *A*_6_ intensity in a larger range (within the repulsive mode). However, larger repulsions may lead to wear, and thus to damage of the tip [21–25].

**Figure 5:**
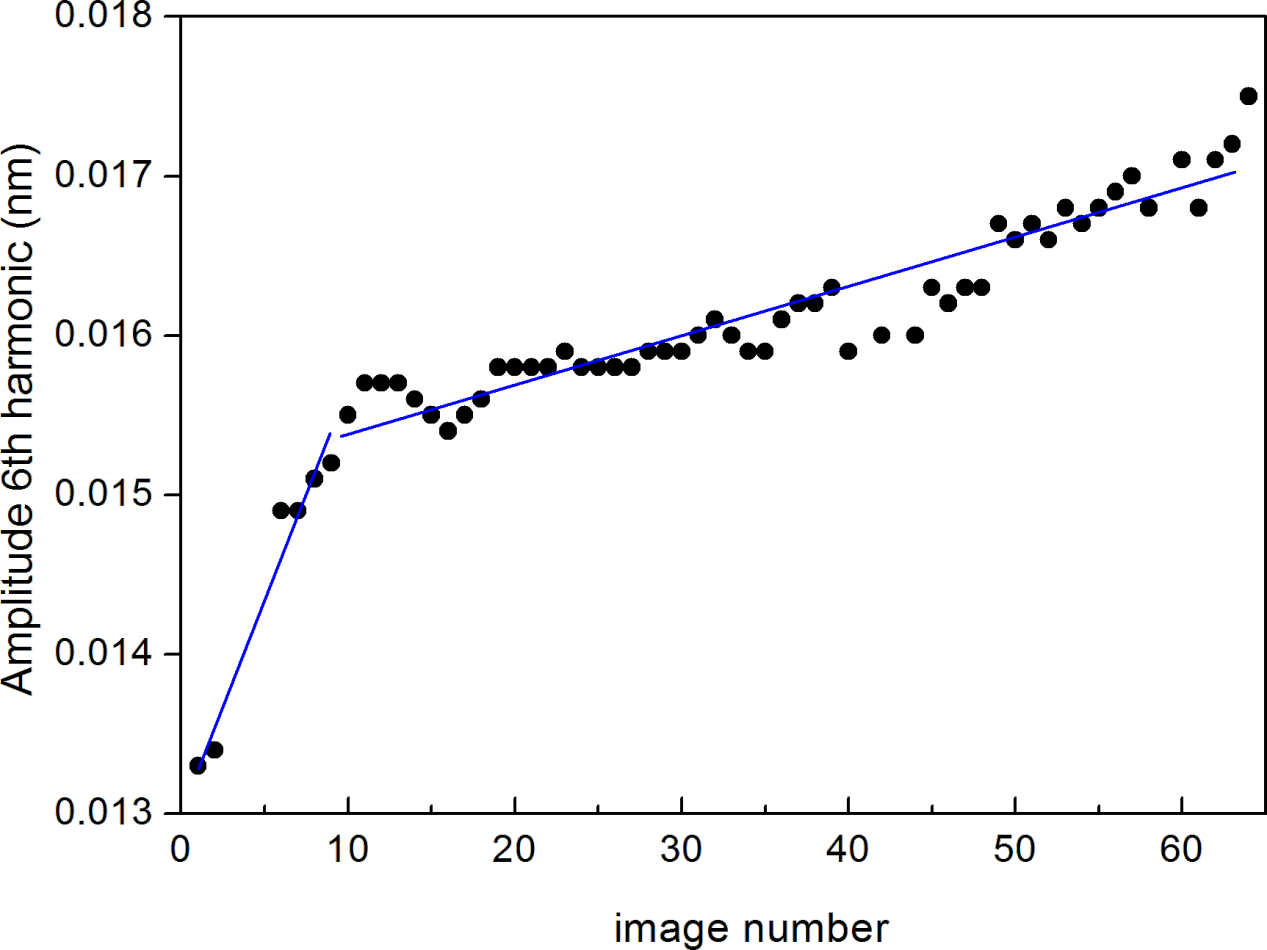
(color online) Evolution of the mean value of the amplitude of the 6th harmonic extracted from the amplitude image simultaneously acquired with the topography and phase images. Experiments have been performed with a nominally 44 N/m rectangular AFM cantilever with resonance frequency 350 kHz on silicon surfaces under ambient conditions. The time evolution is expressed in terms of sequentially acquired images. The free oscillation amplitude was set to 30 nm and setpoint to *A*_sp_ = 27 nm, respectively. The missing points 3, 4 and 5 correspond to approach curves taken for amplitude calibration. The continuous blue lines are guides to the eye.

Additional information can be obtained from the acquired images. Figure 5 shows the evolution of the mean *A*_6_ value acquired simultaneously with the topographic and phase images. As it is observed from the simulations and experiments, the value of *A*_6_ is below 0.1 nm, which implies a very low signal to noise ratio. To overcome this difficulty, *A*_6_ is acquired at each point of the AFM image (256 × 256 points) and then it is calculated by averaging all the values obtained at the image. In this way, the time evolution of the value of *A*_6_ is expressed in terms of sequentially acquired images (each point corresponds to one image), where the experimental parameters such as *A*_1_ and the amplitude setpoint do not change over time. We observe a rapid increase from image 1 to 10 followed by an increase with a smaller slope above image number 10. The figure resembles Figure 2a with a higher slope at the beginning and a lower slope afterwards. Because of the expected tip wear, the evolution observed in Figure 5 can be again ascribed to an increase in tip radius. This method has been proposed to monitor the stability of the tip in a continuous manner [13,26–27].

The quantitative determination of the actual tip radius at each image is a rather difficult task, since it depends critically in several experimental parameters. We have performed a parallel calibration of the tip radius with reference samples. Supporting Information File 1, Figure S2 shows the evolution of *A*_6_ as a function of *R* obtained from commercial gold nanoparticles (5.5 ± 0.7 nm diameters) dispersed on a thin poly-lysine film grown on mica. From the figure we can observe the increase of *A*_6_ for increasing *R* values.

#### Dependence of the amplitude of the 6th harmonic on bulk modulus

**Figure 6:**
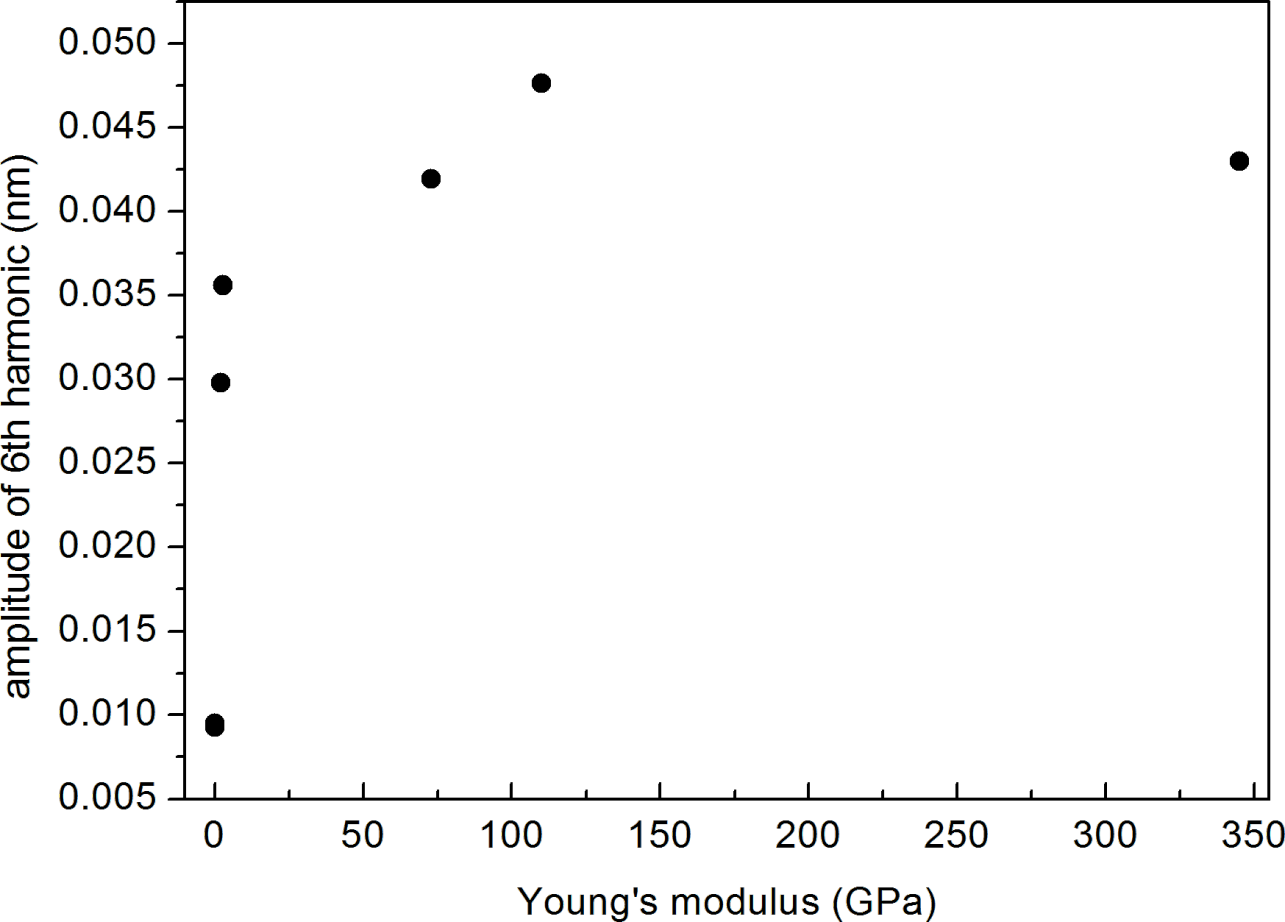
Experimental evolution of *A*_6_ vs *E* for PDMS (*E* = 0.0025 GPa), LDPE (*E* = 0.1 GPa), PS (*E* = 2.7 GPa), fused silica (*E* = 72.9 GPa), titanium (*E* = 110 GPa) and sapphire (*E* = 345 GPa) using a 10.9 N/m cantilever.

Figure 6 shows the evolution of *A*_6_ for discrete values of Young’s modulus from different materials, namely PDMS (*E* = 0.0025 GPa), LDPE (*E* = 0.1 GPa), PS (*E* = 2.7 GPa), fused silica (*E* = 72.9 GPa), titanium (*E* = 110 GPa) and sapphire (*E* = 345 GPa), using a 10.9 N/m cantilever, as determined with the thermal tune method and Sader’s corrections [28].

In spite of the reduced number of experimental points, the curve can be compared to Figure 2b, with a sharp increase at low *E* values and a nearly constant value for larger *E* values. One has to take into account that during the experiments, where the tip has to be retracted and samples have to be changed, a variation of the tip radius cannot be excluded. The sample with the highest wear was titanium, because of its higher roughness as compared to the rest of the samples, and for this reason it was measured at the end of the cycles. From the figure it can be clearly observed that *A*_6_ for the titanium sample shows the larger values.

#### Dependence of the amplitude of the 6th harmonic on free oscillation amplitude

**Figure 7:**
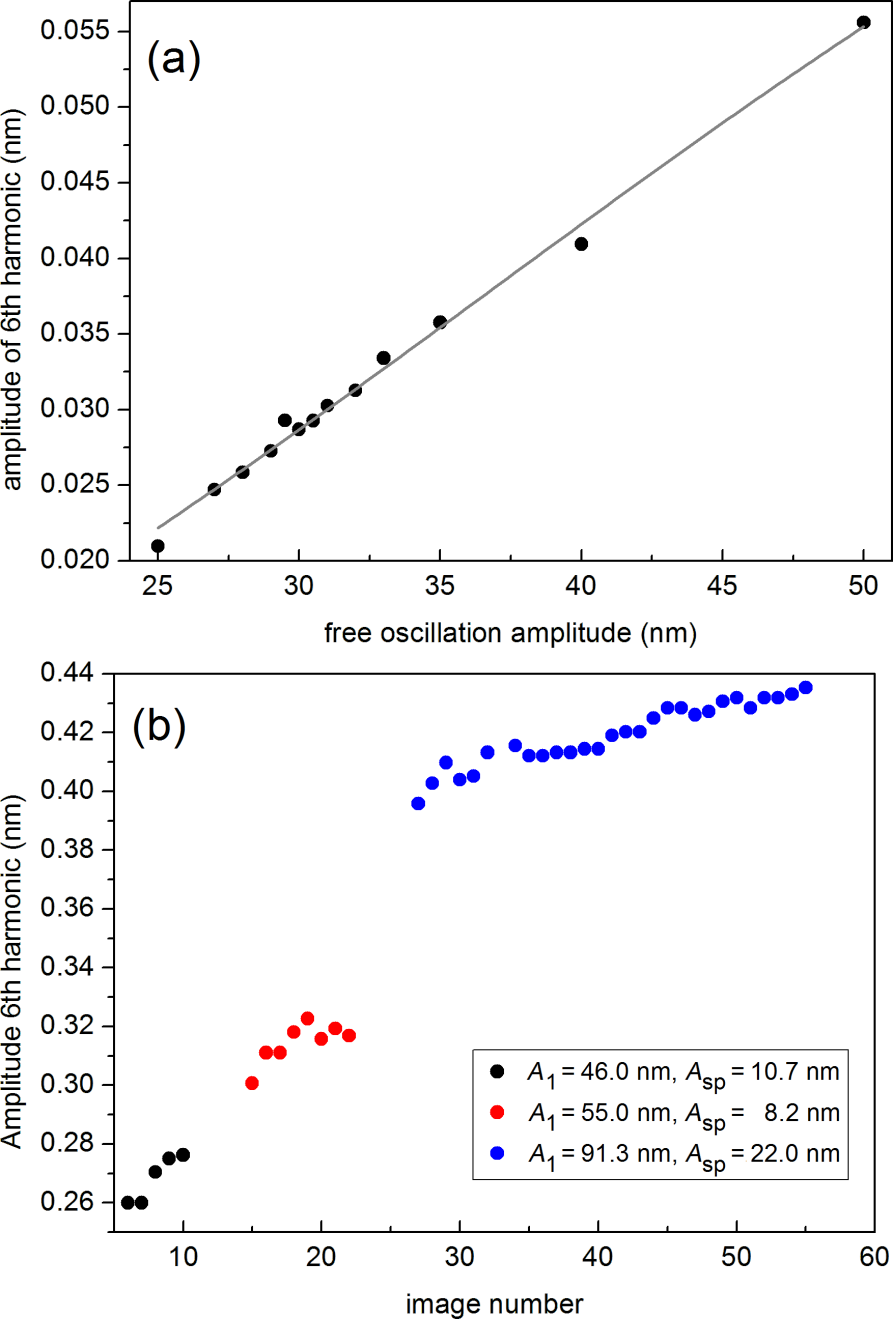
(color online) (a) Experimental evolution of *A*_6_ as a function of the free oscillation amplitude using 26 N/m cantilevers. The fit to Equation 4 leads to *g*_1_ = 3,016.794, *g*_2_ = 38.23 nm^−1^ and *g*_3_ = −569.14 nm^−1/2^. (b) Evolution of the mean value of the amplitude of the 6th harmonic extracted from the amplitude images simultaneously acquired with the topography and phase images. The amplitude has been calibrated using the sensitivity determined from approach curves. The experiments have been performed with a *k*_c_ = 3.1 N/m rectangular AFM cantilever with a resonance frequency of 74.46 kHz and *Q* = 231, as determined with the thermal tune method, on silicon surfaces. The time evolution is expressed in terms of sequentially acquired images. Three different *A*_1_ and *A*_sp_ pairs have been used: *A*_1_ = 46.0 nm, *A*_sp_ = 10.7 nm (black full dots), *A*_1_ = 55.0 nm, *A*_sp_ = 8.2 nm (red full dots) and *A*_1_ = 91.3 nm, *A*_sp_ = 22.0 nm (blue full dots).

Figure 7a shows the evolution of *A*_6_ as a function of the free oscillation amplitude as determined experimentally with nominally 26 N/m cantilevers. The experimental points (full black circles) correspond to the mean *A*_6_ values obtained from the average of a full image acquisition of the 6th harmonic, in analogy to the experiments described in Figure 5. The points have been fitted to the *G* function from Equation 4. The nearly linear behaviour agrees with the predictions shown in Figure 2c and strongly suggests that the tip radius has not changed during the measurements, since otherwise the behaviour of *A*_6_ would have been nonlinear, with a slope increasing for increasing *A*_1_ values.

In addition, Figure 7b shows the evolution of *A*_6_ as a function of sequentially acquired images using softer cantilevers (*k*_c_ ≈ 3 N/m), where both *A*_1_ and *A*_sp_ have been intentionally varied during the experiment. Note that here *A*_6_ increases for increasing *A*_1_ values, as observed in Figure 7a, and that for a particular couple of *A*_1_ and *A*_sp_ values, *A*_6_ increases due to tip wear, and thus to an increase in tip radius.

Note the higher *A*_6_ values in Figure 7b, as compared to those in Figure 7a for similar *A*_1_ values. This is essentially due to the softer cantilevers used in Figure 7b, an effect described in Figure 3.

## Conclusion

Based on numerical simulations using the VEDA code we have proposed a functional dependence of the amplitude of the 6th harmonic of rectangular cantilevers on the tip radius, sample’s Young’s modulus, free oscillation amplitude and cantilever force constant. The 6th harmonic has been chosen because it is in close resonance with the 2nd flexural mode, leading to observable signals, and because its frequency is within the reach of the control electronics used in the experiments. The outcome of the simulations is that the 6th harmonic can be analytically expressed by the product of four identical decoupled gun-shaped functions, each one associated with a specific parameter and with its own coefficients. Thus, the partial evolution for a particular parameter can be traced using this universal behaviour.

The simulations have been validated with AFM experiments using rectangular cantilevers in the 3–45 N/m range and different samples in the 2–345 GPa range. The predicted trends are well reproduced by the experiments. As we can notice from the different trends, the 6th harmonic is most sensitive to changes in tip radius for values of tip radius below ca. 10 nm and Young modulus below ca. 20 GPa. If we consider the method for implementing tip radius real time monitoring, this will be more effective when sharp (new) tips are imaging stiff samples. Instead, in order to measure the surface Young modulus the modelling shows that the best results will be obtained using larger tip radius (10–15 nm) in order to have an almost constant radius-dependent contribution.

So far the results are only qualitative. In order to obtain trustworthy predictions, a more precise and more accurate calibration of the cantilever is still necessary. The performed simulations might also be improved including parameters which could be varied and measured experimentally in order to refine the method and make the results of the simulations more comparable to the experimental ones. The mathematical tool that we present here based on gun-shaped functions allows to identify the optimal conditions to obtain information about the properties of materials from the harmonic response in non-contact dynamic modes.

## Simulations and Experimental Details

### Simulations

Simulations have been performed using the Virtual Environment for Dynamic AFM (VEDA) open code, which takes into account the dynamics of oscillating rectangular cantilevers with multiple eigenmodes [16]. The frequencies, stiffness and quality factors of the 2nd flexural eigenmode have been approximated by the known relationships 6.27*f*, 6.27^2^*k*_c_ and 6.27*Q*, respectively, corresponding to a massless tip [29]. As mentioned above, we have used the DMT model to describe the tip–surface interaction and the tip has been approximated by a hemisphere with a well-defined radius *R*. For simplicity neither viscoelastic nor capillary forces have been considered. The negligible contribution of viscoelastic forces in PS with 25 N/m cantilevers is discussed in the Supporting Information File 1 (see Figure S3).

### Experimental

AFM experiments have been performed with a Bruker DIMENSION ICON instrument hosted in a homemade controlled humidity environment with Nanoscope V control electronics. Commercial rectangular microfabricated silicon cantilevers with ultrasharp silicon tips (*R* < 10 nm) have been used with the following nominal values: OTESPA (Bruker) with *k*_c_ ≈ 44 N/m and 300 ≤ *f*_0_ ≤ 400 kHz, OTESPA-R3 (Bruker) with *k*_c_ ≈ 25 N/m and 200 ≤ *f*_0_ ≤ 400 kHz, and PPP-FMR (Nanosensors) with *k*_c_ ≈ 3 N/m and 70 ≤ *f*_0_ ≤ 80 kHz, where *f*_0_ stands for the resonance frequency. The amplitudes of the higher harmonics were registered using an internal lock-in amplifier. In general, such amplitudes will depend on the selected working frequency. The effect of working slightly off-resonance is discussed in Supporting Information File 1, Figure S4. The estimation of the *A_n_* magnitudes (in nm) has been obtained by calibration of the laser-detector sensitivity, which is about 100 nm/V, as determined from force curves. Humidity was kept below 20%. The *z* motion of the piezoscanner has been calibrated using virtual standards [30]. Due to the value of the fundamental resonance frequency of the employed cantilevers, we have focused in the resonance of the 2nd flexural mode and the 6th harmonic, which frequencies are below 2.5 MHz, since the control electronics is limited to 5 MHz.

## Supporting Information

File 1 Simulated evolution of the phase of the 6th harmonic as a function of tip radius. Correlation between the amplitude of the 6th harmonic and the tip radius obtained from gold nanoparticles dispersed on mica. Simulated evolution of the amplitude of the 6th harmonic as a function of the z distance. Off-resonance experimental approach curves.
