# Supplementary material for: Functional dependence of resonant harmonics on nanomechanical parameters in dynamic mode atomic force microscopy
Source: Beilstein J Nanotechnol. 2017 Apr 19;8:883–91. doi: 10.3762/bjnano.8.90 (PMC5405692; doi:10.3762/bjnano.8.90)
Supplement: File 1 — Simulated evolution of the phase of the 6th harmonic as a function of tip radius. Correlation between the amplitude of the 6th harmonic and the tip radius obtained from gold nanoparticles dispersed on mica. Simulated evolution of the amplitude of the 6th harmonic as a function of the z distance. Off-resonance experimental approach curves. [file Beilstein_J_Nanotechnol-08-883-s001.pdf]

Supporting Information

for

Functional dependence of resonant harmonics on nanomechanical parameters in dynamic mode atomic force microscopy

Federico Gramazio[1], Matteo Lorenzoni[2], Francesc Pérez-Murano[2], Enrique Rull Trinidad[3], Urs Staufer[3] and Jordi Fraxedas*[1]

Address: [1]Catalan Institute of Nanoscience and Nanotechnology (ICN2), CSIC and The Barcelona Institute of Science and Technology, Campus UAB, Bellaterra, 08193 Barcelona, Spain, [2]Instituto de Microelectrónica de Barcelona (IMB-CNM, CSIC), Campus UAB, 08193 Bellaterra, Barcelona, Spain and [3]Technical University of Delft, Mekelweg 2, 2628CD Delft, The Netherlands

Email: Jordi Fraxedas - jordi.fraxedas@icn2.cat

* Corresponding author

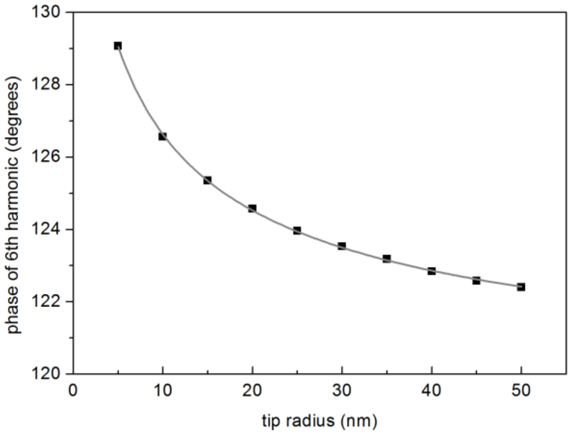

Figure S1: Simulated evolution of the phase of the 6th harmonic (full black squares) as a function of the tip radius. The calculations have been performed for a silicon rectangular cantilever with k_c = 25 N/m, f_0 = 300 kHz and Q = 400 with A_1 = 30 nm, R = 10 nm, E = 3GPa (as in Figure 1) and an amplitude setpoint of 10.8 nm (z = 10 nm). The continuous grey line corresponds to a least-square fit using the gun-shape function from Equation 4: g_1 = 0.00138 nm·degrees^{-1}, g_2 = 0.00845 degrees^{-1} and g_3 = −0.00218 nm$^{1/2}$·degrees^{-1}.

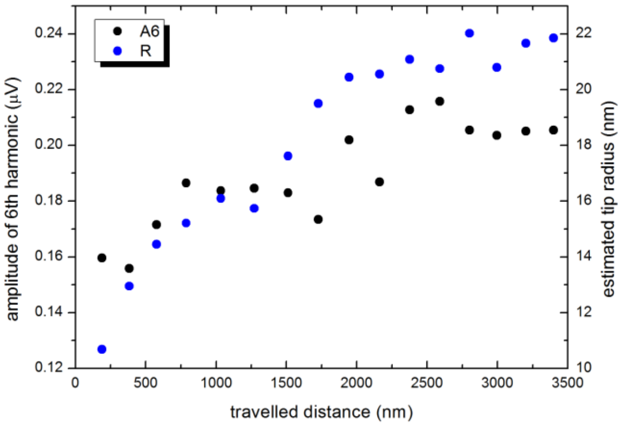

Figure S2: Evolution of the amplitude of the 6th harmonic (full black circles) and of the estimated tip radius (full blue circles) as a function of the distance travelled by the tip.

We have acquired AFM images of topograhy and amplitude of the 6th harmonic on a calibration sample of gold nanoparticles, with a diameter of 5.5 ± 0.7 nm, dispersed on a thin layer of poly-lysine deposited on a mica substrate. The estimation of the tip radius has been extracted from the topography images using the geometrical model from Vesenka, J.; Manne, S.; Giberson, R.; Marsh, T.; Henderson, E. Colloidal gold particles as an incompressible atomic force microscope imaging standard for assessing the compressibility of biomolecules. *Biophysical Journal* **1993**, *65*, 992–997.

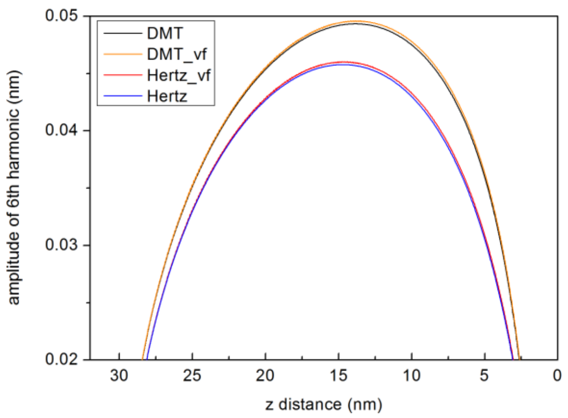

Figure S3: Simulated evolution of the amplitude of the 6th harmonic as a function of the *z* distance. Both DMT and Hertz models have been used with no viscoelastic forces (continuous black and blue lines, respectively) and including viscoelastic forces (continuous orange and red lines, respectively). We have used the three element model to account for the viscoelastic forces: E_1 = 3 GPa, E_2 = 11.5 GPa and η = 58 GPa·s, where E_1, E_2 and η stand for the PS Young's modulus, delayed Young's modulus and viscosity, respectively [Cheng, L.; Xia, X.; Scriven, L. E.; Gerberich, W. W. *Spherical-tip indentation of viscoelastic material*, *Mechanics of Materials* **2005**, *37*, 213–226].

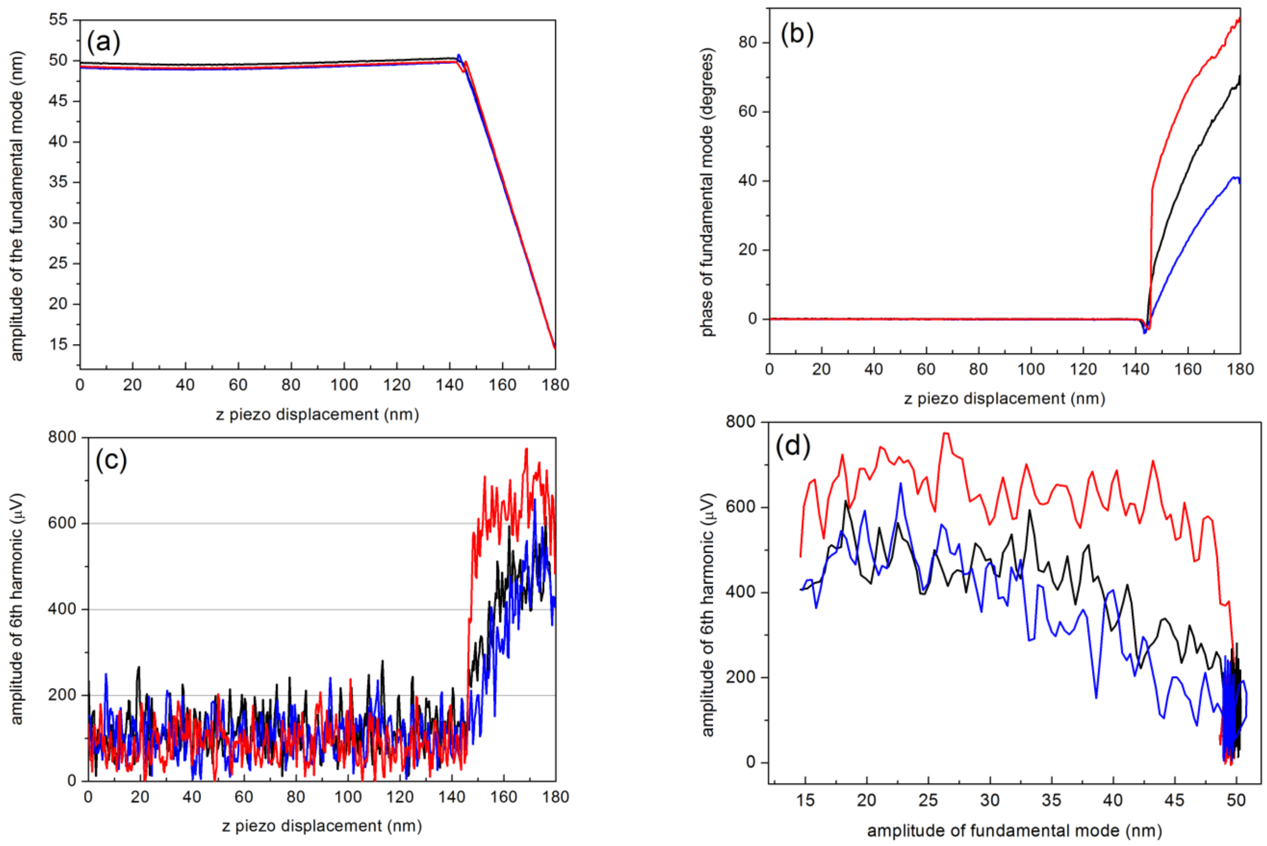

Figure S4: Experimental approach curves: (a) amplitude and (b) phase of the fundamental mode and (c) amplitude of the 6th harmonic as a function of the *z* piezo displacement using cantilevers force constant around 25 N/m with resonance frequencies of about 246 kHz and clean silicon samples. The approach curves were taken when the excitation

frequency corresponds to the frequency of the first eigenmode (continuous black lines), and off-resonance at frequencies corresponding to 90% of the maximum of amplitude, at both sides, below (continuous blue line) and above (continuous red line) resonance, respectively. The individual phase curves have been shifted to 0 degrees (free oscillation) for the sake of comparison. The amplitude of the 6th harmonic as a function of their corresponding amplitudes of the fundamental modes is shown in (d).

From Figure S4c we observe an asymmetric behaviour of the 6th harmonic: above resonance the amplitude is higher. Such an enhancement is due to the fact that, under the experimental conditions and for the particular cantilever used, the frequency of the 6th harmonic is closer to the frequency of the maximum amplitude of the 2nd flexural mode. In Figure S4d we plot the amplitude of the 6th harmonic as a function of their corresponding amplitudes of the fundamental modes. In the absence of tip–surface interaction (free oscillation) we obtain an estimation of the noise level (about ±100 μV). Within the repulsive mode, the amplitude of the 6th harmonic increases for decreasing amplitudes of the fundamental mode (equivalent to the decrease of the amplitude setpoint).
